# Second trimester inflammatory and metabolic markers in women delivering preterm with and without preeclampsia

**DOI:** 10.1038/s41372-018-0275-8

**Published:** 2018-12-05

**Authors:** Kharah M. Ross, Rebecca J. Baer, Kelli Ryckman, Sky K. Feuer, Gretchen Bandoli, Christina Chambers, Elena Flowers, Liang Liang, Scott Oltman, Christine Dunkel Schetter, Laura Jelliffe-Pawlowski

**Affiliations:** 1 Department of Psychology, University of California Los Angeles, Los Angeles, CA USA; 2 Department of Pediatrics, University of California San Diego, San Diego, CA USA; 3 Department of Epidemiology, University of Iowa, Iowa City, IA USA; 4 Department of Obstetrics, Gynecology and Reproductive Sciences, University of California San Francisco School of Medicine, San Francisco, CA USA; 5 California Preterm Birth Initiative, University of California San Francisco, San Francisco, CA USA; 6 Department of Physiological Nursing, University of California School of Nursing, San Francisco, CA USA; 7 Department of Genetics, Stanford University School of Medicine, Palo Alto, CA USA; 8 Department of Epidemiology and Biostatistics, University of California San Francisco School of Medicine, San Francisco, CA USA

**Keywords:** Predictive markers, Endocrine system and metabolic diseases

## Abstract

**Objective:**

Inflammatory and metabolic pathways are implicated in preterm birth and preeclampsia. However, studies rarely compare second trimester inflammatory and metabolic markers between women who deliver preterm with and without preeclampsia.

**Study design:**

A sample of 129 women (43 with preeclampsia) with preterm delivery was obtained from an existing population-based birth cohort. Banked second trimester serum samples were assayed for 267 inflammatory and metabolic markers. Backwards-stepwise logistic regression models were used to calculate odds ratios.

**Results:**

Higher 5-α-pregnan-3β,20α-diol disulfate, and lower 1-linoleoylglycerophosphoethanolamine and octadecanedioate, predicted increased odds of preeclampsia.

**Conclusions:**

Among women with preterm births, those who developed preeclampsia differed with respect metabolic markers. These findings point to potential etiologic underpinnings for preeclampsia as a precursor to preterm birth.

## Introduction

Preeclampsia is a gestation-specific syndrome, defined by the presence of hypertension combined with proteinuria or evidence of systemic disease that presents after 20 weeks gestational age (GA) [1]. Affecting 2−8% of pregnancies, preeclampsia is a major cause of maternal mortality [2]. The only current treatment is induction of labor, resulting in increased risk for preterm birth, perinatal death, and neonatal complications [2].

Although the etiology of preeclampsia is still poorly understood, it is considered to be a vascular disorder caused by distress signals released by an ischemic placenta [1]. Similar to nonpregnancy vascular and cardio-metabolic disorders, e.g. atherosclerosis and metabolic syndrome, preeclampsia is believed to have etiological roots in inflammatory and metabolic disturbances [1]. Metabolomic analyses of blood and urine collected from women with preeclampsia versus healthy pregnancies have identified several potential metabolic pathways implicated in preeclampsia, including insulin resistance, mitochondrial dysfunction, energy metabolism disturbances, oxidative dysfunction and lipid dysfunction [3–15]. Although less studied, the evidence supports preeclampsia as a proinflammatory state [16–23]. However, most studies of inflammatory activity in preeclampsia have been conducted either following diagnosis or, if prior, they examined only a few inflammatory markers (e.g. C-reactive protein, interleukin (IL) 6, neutrophil gelatinase-associated lipocalin, tumor necrosis factor (TNF) α, vascular cellular adhesion molecule-1) [18, 19] (for exceptions, see refs. [21, 24]).

Similar to preeclampsia, preterm birth, i.e. <37 weeks GA, could have roots in metabolic and inflammatory disturbances, e.g. placental damage or vascular lesions [25]. Previous studies have focused on differences in metabolic and inflammatory activity between women with healthy pregnancies and women with either preeclampsia *or* who delivered preterm. Few studies have assessed early pregnancy inflammatory and metabolic differences between women who delivered preterm and who delivered preterm with preeclampsia. Indeed, only one study was found that examined metabolic differences between four women who developed preeclampsia and five women who delivered preterm. Distinct second trimester serum phospholipid profiles by pregnancy outcome were observed, but the study (*n* = 9) was not sufficiently powered to characterize those differences [14]. No studies were found that evaluated inflammatory marker differences between women who delivered preterm with or without preeclampsia. Investigating the early pregnancy differences in metabolic and inflammatory markers between women who later delivered preterm with or without preeclampsia could contribute to our understanding the inflammatory and metabolic etiologic divergences between these adverse pregnancy outcomes.

The aim of this study was to examine differences in mid-pregnancy inflammatory and metabolic markers between women who delivered preterm (<37 weeks GA) with or without preeclampsia. Given differing phenotypes, we hypothesized that women with preterm birth and preeclampsia would exhibit different immune and metabolic profiles compared to GA-matched women delivering preterm without preeclampsia.

## Methods

### Preeclampsia and non-preeclampsia groups

The sample of 129 women was drawn from a population-based cohort of singleton California births (July 2009 to December 2010), which is described in detail elsewhere [26]. Briefly, all women had mid-pregnancy nonfasting serum samples (15−20 weeks’ gestation) banked by the California Biobank Program following protocols used for routine prenatal screening for aneuploidies and neural tube defects by the California Genetic Disease Screening Program. Fetuses with a known congenital anomaly were excluded from the analysis. Detailed demographic and obstetric information were obtained from a linked hospital discharge birth cohort database maintained by the California Office of Statewide Health Planning and Development (OSHPD). The final source set for this study included 496 women with deliveries before 37 weeks, with fortification for deliveries before 32 weeks.

Within this cohort, 43 women delivered preterm (<37 weeks GA) and had a diagnosis of preeclampsia in the hospital discharge record for their delivery. International Classification of Diseases, Ninth Revision, Clinical modification (ICD-9-CM) criteria was used for coding (642.4 (mild), 642.5 (severe), and 642.7 (superimposed on preexisting hypertension)). These cases were matched by GA at a ratio of 2:1 with 86 women who delivered preterm but without a preeclampsia diagnosis.

### Serum inflammatory and metabolic markers

Banked serum samples were stored in 1 mL tubes at −80 °C prior to assay. Metabolic profiling was performed by Metabolon, Inc., a commercial supplier of global metabolomics data. Briefly, three sample extracts were used for chromatography/mass spectrometry (LC/MS, Waters ACQUITY UPLC and Thermo-Finnigan LTQ mass spectrometer) in (1) positive (acidic) and (2) negative (basic) ionization modes, as well as (3) gas chromatography/mass spectrometry (GC/MS, Thermo-Finnigan Trace DSQ fast-scanning single-quadrupole mass spectrometer). Peaks were identified via Metabolon’s propriety peak integration software and compounds were identified through comparison of library entries of purified standards or recent unknown entities, with data normalization to correct any variation due to instrument inter-day tuning differences. Metabolic marker concentrations were transformed by dividing by the cohort sample median for that metabolite. As such, values less than “1” indicate metabolite concentrations less than the cohort median. Given previous work documenting preeclampsia-associated disturbances in lipids, carbohydrates and energy metabolites, we focused on 198 metabolites [3–15].

Samples were assayed for 63 inflammatory markers (including interleukins, interferons, chemokine ligands, TNFα family cytokines, growth factors, colony-stimulating factors, soluble adhesion molecules) at the Human Immune Monitoring Center (HIMC; Stanford University), using human multiplex kits (custom-built by eBioscience, San Diego, CA) and read using a Luminex 200 instrument (Austin, TX) in accordance with the manufacturer’s recommendations. Protocol details have been described elsewhere [27]. Median fluorescence intensity values were reported for all inflammatory markers using Masterplex software (Hitashi Solutions, San Bruno, CA). All inter-assay coefficients (CVs) were <15% across all markers, and all intra-assay CVs were <10%.

Adiponectin, leptin, and a series of lipids (total cholesterol, low-density lipoprotein (LDL), high-density lipoprotein, and triglycerides) were assayed at the State Hygienic Laboratory (University of Iowa) using a Roche Diagnostics c111 Cobas Analyzer (Basel, Switzerland). Total cholesterol and LDL levels were converted to multiples of the median. A total of 267 inflammatory and metabolic markers were available for analysis (Supplemental Table 1).

### Covariates

Maternal characteristics were included as covariates, and were obtained from birth certificate record and hospital discharge ICD-9 records. Characteristics obtained from birth certificate records included: race or ethnicity (Black, Hispanic, Asian or “other” race/ethnicity versus White non-Hispanic), maternal age at delivery (“less than 18 years” or “more than 34 years” versus “18 to 34 years”), maternal education (“less than 12 years” or “more than 12 years” versus “12 years or high school”), payment for birth (public insurance for birth (Medi-Cal, California’s Medicaid, health coverage for low-income persons) versus not), parity (nulliparous versus multiparous), nativity (maternal birthplace outside the US versus within the US), smoked during pregnancy (report of any smoking versus no report), previous preterm birth (previous preterm birth versus none), and prepregnancy body mass index (BMI). Maternal BMI was calculated from prepregnancy weight and height. Women were categorized as underweight (less than 18.5 kg/m^2^), normal (18.5−24.9 kg/m^2^), overweight (25.0−29.9 kg/m^2^), obese (30.0 kg/m^2^ or more) compared to normal BMI (18.5−24.9 kg/m^2^), with “normal” BMI being the comparison group.

Maternal factors obtained from hospital discharge ICD-9 records included diabetes, drug or alcohol dependence/abuse, mental illness, and anemia. Women were coded as having “diabetes” if they had ICD-9 codes indicating any form of diabetes or abnormal glucose tolerance (ICD-9s 648.0—Diabetes mellitus complicating pregnancy, childbirth or the puerperium, 250—Diabetes mellitus, 648.8—Abnormal glucose tolerance complicating pregnancy, childbirth or the puerperium) and were compared to women with no diabetes. Drug or alcohol dependence was indicated in the presence of any ICD-9 code indicating drug or alcohol use (ICD-9s 648.3—Drug dependence complicating pregnancy, childbirth or the puerperium; 304—Drug dependence; 305—Nondependent abuse of drugs, 303—Alcohol dependence syndrome), and were compared to women with no indication of drug or alcohol abuse. Women were coded as having a mental illness (ICD-9 code 648.4—Mental disorder complicating pregnancy, childbirth or the puerperium) versus no mental illness. And women were coded as having anemia (648.2—Anemia complicating pregnancy, childbirth or the puerperium) versus not.

### Analytic strategy

First, logistic regression was used to compare women with and without preeclampsia on maternal characteristics and each of the inflammatory and metabolic markers. Crude odds ratios (OR) and 95% confidence intervals (95% CI) were calculated (Supplemental Table 1). Maternal characteristics and biomarkers with crude ORs of *p* < 0.2 were retained for additional model building (see below).

Next, a multivariable model for preeclampsia was built using backwards-stepwise logistic regression to calculate OR for each maternal covariate and biomarker. All variables with *p* < 0.20 in initial crude analyses were included in the first step of the model. The backwards-stepwise logistic regression then sequentially removes variables in order of greatest *p*-value, re-running the model after each variable is removed. Removal of variables continues until no additional variables can be removed without a significant loss of model fit, and the final model included only variables with a *p* < 0.05. Power analyses indicated that, given our sample size and a logistic regression approach, our analyses are capable of detecting ORs > 2.29 or ORs < 0.436 [27]. A sample was created using cross-validation with replacement using a replicate size of 10 to evaluate the replicability of the multivariable model.

## Results

### Sample characteristics

Sample characteristics are presented in Table 1. Participants were on average 29.9 ± 6.29 years old and predominately Latina/Hispanic (52%) or non-Latina White/Caucasian (33%). Approximately half (49%) of the sample were receiving MediCal insurance. Women on average gave birth at 32.4 ± 3.6 weeks GA. Women diagnosed with preeclampsia had marginally higher prepregnancy BMI, *t*(127) = −1.92, *p* = 0.057 (27.6 ± 7.57 kg/m^2^ versus 25.3 ± 5.85 kg/m^2^). There were no group differences at *p* < 0.05.

**Table 1:** Sample characteristics

Variable	Full sample (*n* = 129)	PE (*n* = 43)	Non-PE (*n* = 86)	*p*
Race/ethnicity				
White	33% (43)	37% (16)	31% (27)	0.401
Latina	52% (67)	56% (24)	50% (43)	
Asian	9% (12)	5% (2)	12% (10)	
Black	2% (2)	0% (0)	2% (2)	
Unknown	4% (5)	2% (1)	5% (4)	
Age (years)	29.9 ± 6.29	30.4 ± 6.60	29.6 ± 6.15	0.490
Public insurance	49% (63)	44% (19)	51% (44)	0.455
Prepregnancy BMI	26.1 ± 6.53	27.6 ± 7.57	25.4 ± 5.85	0.057
GA at assessment (weeks)	16.4 ± 1.01	16.5 ± 1.10	16.3 ± 0.963	0.461
GA at birth (weeks)	32.4 ± 3.64	32.4 ± 3.67	32.4 ± 3.64	1.00
Delivery mode (C-section)	61% (79)	78% (33)	53% (46)	0.011

### Model building

Three maternal characteristics differed (*p* < 0.2) between women with and without preeclampsia and were included as covariates in the multivariable model: mother born outside of the United States, OR = 0.4, 95% CI (0.2, 0.9), obese BMI, OR = 2.3, 95% CI (1.0, 5.7), and diabetes, OR = 2.3, 95% CI (1.0, 5.1). Of the biomarkers examined, two inflammatory markers and 32 metabolic markers differed between women with and without preeclampsia (*p* < 0.2) and were also entered in the multivariable model (Supplemental Table 1).

The final multivariable model included two significant maternal covariates: Women born outside of the United States were at lower odds of developing preeclampsia, adjusted OR = 0.43, *p* = 0.048, 95% CI (0.19, 0.99), while women with diabetes were at higher odds, adjusted OR = 2.80, 95% CI (1.14, 6.85). With respect to biomarkers, of the two inflammatory and 32 metabolic markers, only four metabolic markers significantly differentiated between women who delivered preterm with or without preeclampsia independent of covariates. Specifically, higher levels of 1-linoleoylglycerophosphoethanolamine, adjusted OR = 0.29, *p* = 0.007, 95% CI (0.12, 0.71), and octadecanedioate, OR = 0.27, *p* = 0.021, 95% CI (0.09, 0.82), were associated with reduced odds of preeclampsia. Elevated levels of 5-α-pregnan-3β,20α-diol disulfate, adjusted OR = 2.74, *p* = 0.020, 95% CI (1.17, 6.38), and 5-HETE, adjusted OR = 1.30, *p* = 0.004, 95% CI (1.09, 1.54), were associated with an increased odds of preeclampsia (Table 2).

**Table 2:** Multivariable model predicting pregnancy outcome (preterm birth with or without preeclampsia) from the four mid-pregnancy serum metabolic markers and adjusting for diabetes diagnosis and birth outside the US

Maternal factor	Source sample		Cross-validation sample	
	aOR (95% CI)	*p* value	aOR (95% CI)	*p* value
Mother born outside US	0.43 (0.19, 0.99)	0.048	0.61 (0.35, 1.54)	0.298
Diabetes	2.80 (1.14, 6.85)	0.025	2.66 (0.91, 7.76)	0.073
1-linoleoylglycerophosphoethanolamine	0.29 (0.12, 0.71)	0.007	0.32 (0.12, 0.85)	0.022
Octadecanedioate	0.27 (0.09, 0.82)	0.021	0.32 (0.09, 1.06)	0.062
5-α-pregnan-3β,20α-diol disulfate	2.74 (1.17, 6.38)	0.020	2.72 (1.07, 6.95)	0.036
5-HETE	1.30 (1.09, 1.54)	0.004	1.14 (0.92, 1.41)	0.238

In the cross-validation sample, of the two covariates, only diabetes diagnosis predicted greater odds of a preeclampsia. With respect to the four metabolic markers, only three remained significant in the cross-validation sample. Similar to the source sample, higher levels of 1-linoleoylglycerophosphoethanolamine and octadecanedioate predicted reduced odds of preeclampsia, and higher levels of 5-α-pregnan-3β,20α-diol disulfate predicted higher odds of preeclampsia (Table 2). In the cross-validation sample, 5-HETE was not significantly associated with odds of developing preeclampsia (Table 2).

## Discussion

The purpose of this study was to compare second trimester metabolic and inflammatory factors in preterm births with and without preeclampsia. Independent of maternal characteristics, we found that women who delivered preterm with or without preeclampsia were more similar than different in terms of inflammatory and metabolic profiles at 15−20 weeks gestation. However, of the 198 metabolic markers, three emerged as differentiators between women who did or did not develop preeclampsia. Specifically, elevated second trimester serum levels of 5-α-pregnan-3β,20α-diol disulfate, a progesterone metabolite, was associated with higher odds of preeclampsia, and higher levels of 1-linoleoylglycerophosphoethanolamine, a lysophospholipid, and octadecanedioate, a long fatty acid chain, with reduced odds of preeclampsia.

Our study found that lower levels of 1-linoleoylglycerophosphoethanolamine in serum was associated with greater odds of a preeclampsia. This finding is consistent with previous research that reported significantly lower levels of lysophospholipids in women who developed preeclampsia compared to healthy, term pregnancies [28]. Lysophospholipids are lipid derivatives with one or both acyl derivatives removed through hydrolysis, and the lysophospholipid system plays a role in cell-to-cell signal transmission and regulates several aspects of reproduction and parturition, including trophoblast function and placentation [29]. Lower levels of 1-linoleoylglycerophosphoethanolamine in second trimester serum in women who develop preeclampsia may suggest early lysophospholipid system dysregulation.

Higher levels of the progesterone metabolite, 5α-pregnan-3β, 20α-diol disulfate, during the second trimester were associated with increased odds of developing preeclampsia. This is consistent with past work that reported higher progesterone levels in the placenta and serum of women with preeclampsia, or who developed preeclampsia, compared to healthy, term controls [30–33] (for exceptions, see refs. [34, 35]). When healthy placentas are exposed to the levels of progesterone obtained from placentas of women with preeclampsia, pathways that promote vasoconstriction in the placenta are activated [33], suggesting that increased progesterone metabolites indicates increased early vasoconstriction in placentas of women who go on to develop preeclampsia. As such, increased serum progesterone metabolites may be an early indicator of placental pathology in women who go on to develop preeclampsia.

In both the source sample and cross-validation sample, lower levels of octadecanedioate were associated with an increased odds of preeclampsia. Octadecanedioate is a fatty acid not commonly found in humans [36]. One study reported that octadecandioate has both antioxidant and antidiabetic properties [37], which is consistent with the association observed here between higher levels of octadecanediote and reduced risk for preeclampsia. No other studies that we are aware of have considered octadecanedioate in the context of pregnancy. Future research is needed to determine how octadecanedioate levels in serum might related to the etiology of preeclampsia in the context of preterm birth.

We note that group differences were observed for metabolic markers but not for inflammatory markers. These data suggest that differences in metabolism may be the key distinguishing processes between women who do and do not develop preeclampsia in women with preterm birth. These data also support previous research showing the inflammation represents a common pathway in the emergence of preterm birth with and without preeclampsia [24]. It is also possible that previously reported associations between preeclampsia and greater inflammation may be due to sample collection *after* preeclampsia diagnosis [16–23], reflecting a secondary symptom of advanced preeclampsia pathology rather than an etiological cause.

While this study is among the first to compare early pregnancy differences in combined inflammatory and metabolic activity between women who later deliver preterm with or without preeclampsia, it has some limitations. First, we had access to samples from only one time point in pregnancy. There is evidence that inflammatory and metabolic activity changes over the course of pregnancy [38, 39]. While we captured mean-level differences early in pregnancy, it is also possible that rate of or lack of change in inflammatory and/or metabolic markers could be indicative of early pathological processes [19]. Examining changes in markers over pregnancy may help differentiate between women who develop preeclampsia versus those who do not. Second, our sample was taken from a retrospective cohort study, and we were restricted to data available in linked medical records. Potentially valuable information, e.g. onset, severity and history of preeclampsia, or type and cause of preterm birth, was either not available or we were too underpowered to consider these additional factors. Additional research in larger samples is needed to understand how additional medical factors may be related to early pregnancy inflammatory and metabolic activity. Finally, due to our primary interest in etiological differences between women who delivered preterm with or without preeclampsia, a control group of women with healthy pregnancies was not included. Future research should explore how similarities or differences in mid-pregnancy inflammatory and metabolic markers between women who delivered preterm with or without preeclampsia compared to healthy pregnancies.

In summary, while research has shown that inflammatory and metabolic activity is involved in early etiology of both preeclampsia and preterm birth in general [3–23, 25], no studies to our knowledge have directly assessed potential differences across these pathways in women who deliver preterm with and without preeclampsia. Of the 267 metabolic and inflammatory markers tested, only three were associated with risk for preeclampsia in women with preterm birth, suggesting that overall women who deliver preterm with and without preeclampsia have fairly similar metabolic and inflammatory profiles in mid-pregnancy. Risk for preeclampsia was, however, associated with metabolic markers related to progesterone, lysophospholipid and fatty acid metabolism, which may play an etiologic role in the development of preeclampsia in women with preterm birth. While findings require further investigation, these data could allow for the early identification of women at risk for preterm birth with preeclampsia and for further inquiry into pathways that may be amenable to intervention.

## Disclaimer

Data from the California Prenatal Screening Program were obtained through the California Biobank Program (Screening Information System request no. 476). Data were obtained with an agreement that the California Department of Public Health is not responsible for the results or conclusions drawn by the authors of this publication. The funding sources had no role in study design, collection, analysis or interpretation of the data, writing the report, or the decision to submit the article for publication.
